# Effect of Reinforcement Size on Mechanical Behavior of SiC-Nanowires-Reinforced 6061Al Composites

**DOI:** 10.3390/ma15238484

**Published:** 2022-11-28

**Authors:** Qiqi Zhao, Boyu Ju, Keguang Zhao, Junhai Zhan, Mingda Liu, Ningbo Zhang, Jinrui Qian, Ziyang Xiu, Pengchao Kang, Wenshu Yang

**Affiliations:** 1 School of Materials Science and Engineering, Harbin Institute of Technology, Harbin 150001, China; 2 Huazhong Institute of Electro-Optics, Wuhan 430074, China; 3 Shanghai Aerospace System Engineering Research Institute, Shanghai 201108, China; 4 CASIC Space Engineering Development Co., Ltd., Xinzhou 431400, China; 5 Aerospace Research Institute of Materials & Processing Technology, Beijing 100076, China

**Keywords:** SiC nanowires, Al matrix composite, mechanical characterization, interface

## Abstract

In the present study, the effects of SiC nanowires (SiCnws) with diameters of 100 nm, 250 nm and 450 nm on the microstructure and mechanical behavior of 20 vol.% SiCnws/6061Al composites prepared by pressure infiltration were studied. It was found that the interface between SiCnws and Al matrix was well bonded, and no interface product was found. The thicker SiCnws are beneficial to improve the density. In addition, the bamboo-like and bone-like morphologies of SiCnws produce a strong interlocking effect between SiCnws and Al, which helps to improve the strength and plasticity of the material. The tensile strength of the composite prepared by SiCnws with a diameter of 450 nm reached 544 MPa. With a decrease in the diameter of SiCnws, the strengthening effect of SiCnws increases. The yield strength of SiCnws/6061Al composites prepared by 100 nm is 13.4% and 28.5% higher than that of 250 nm and 450 nm, respectively. This shows that, in nano-reinforced composites, the small-size reinforcement has an excellent improvement effect on the properties of the composites. This result has a guiding effect on the subsequent composite structure design.

## 1. Introduction

SiC nanowires have the advantages of high strength, strong oxidation resistance, good wear resistance, wide band gap, excellent electromagnetic wave absorption performance, and high temperature stability. They have potential applications in field emitters, electromagnetic wave absorbers, microelectronics and light emitters, ceramics and metal composites [1,2,3,4]. Studies have shown that polymer [5,6], ceramic [7,8,9,10] and C/C-based [11,12] composites reinforced by SiC nanowires (SiCnws) exhibit higher strength, toughness and thermal shock resistance.

Currently, the use of SiCnws-reinforced metal matrix composites has also been reported. Li et al. [13] prepared 0.3 wt.% SiCnws/Mg-2Zn-0.1Y composite, and the yield strength and tensile strength of the material reached 495.53 MPa and 514.18 MPa, respectively. Zhang et al. [14] added SiCnws with a diameter of 100–600 nm to AZ91D. The results showed that SiCnws had a good combination with the Mg matrix, and the yield strength of the material increased by 33%. Liu et al. [15] reported that the addition of 0.5 wt.% SiCnws (100–200 nm in diameter) to the Ti matrix increased the tensile strength by 52% due to grain refinement and the bridging, drawing and fracture of SiCnws. Li et al. [16] found that the fracture toughness of 20 wt.% SiC whisker (diameter 1.5 μm) reinforced SiC composites increased by 35.5% compared with the matrix. K. Komai et al. [17] prepared SiCnws/7075Al composites (SiCnws diameter range and average size are 0.1–1 μm, 0.5 μm, respectively) by powder metallurgy method. The results show that the properties of SiCnws/7075Al composites are higher than those of the matrix aluminum alloy except for the elongation of fatigue fracture. Jintakosol et al. [18] prepared 5–15 vol.% SiCnws/Al composites, indicating that the wear resistance of the material increases with the increase in SiCnws content.

Our group reported in detail the microstructure and mechanical behavior of SiCnws (diameter distribution 50~800 nm, average diameter ~220 nm) reinforced pure aluminum [19,20], 6061Al [21,22,23] and 2024Al [24] matrix composites. The results show that the interface between SiCnws and Al exhibits good interfacial bonding, and there are no Al_4_C_3_, Al_2_O_3_ and MgAl_2_O_4_ products at the interface [20,25]. The composites show good machinability, the grain of Al matrix in 15 vol.% SiCnws/6061Al composites is obviously refined, and the material shows high strength while retaining the plasticity of aluminum [23]. Adin et al. [26,27] found that the type, content and morphology of reinforcements have an important influence on the properties of composites. When the average size of SiCnws is the same and the content of bamboo-like SiCnws increases from 14.6% to 45.3%, the tensile strength and elongation increase by 8% and 57%, respectively [22]. It is well known that the strengthening effect of particle-reinforced composites is closely related to the size of the reinforcing phase. The smaller the particle size, the more obvious the strengthening effect. In nano-reinforced composites, when the size of the nano-reinforcement is close to the microstructure, such as dislocations and grain boundaries in the matrix, a new phenomenon that is rare in traditional micron reinforcements can be produced [28,29,30,31,32,33]. However, the influence of diameter size on the strengthening behavior of nanowires-reinforced metal matrix composites is not clear, and the structural design and preparation of composites with different diameters lack theoretical guidance. At the same time, for the reinforced phase with a size of 1–100 nm, there are few reports on the preparation of composites with a volume fraction greater than 10% [13,15,34,35,36,37].

In this study, SiCnws with an average diameter of 100 nm, 250 nm and 450 nm were used as reinforcements to study the effect of nanowire size on the microstructure and mechanical behavior of 20 vol.% SiCnws/6061Al composites prepared by pressure infiltration.

## 2. Materials and Methods

### 2.1. Materials and Composite Fabrication Process

In this experiment, SiC nanowires with average diameters of 100 nm, 250 nm and 450 nm were used as raw materials, abbreviated as 100D, 250D and 450D (where ‘D’ represents diameter). SiCnws of 100D were prepared using the thermal evaporation of silicon powder method, in which silicon powder (≥99.0%, <5 μm, Beijing Xing Rong Yuan Technology Co., Ltd., Beijing, China) was used as the silicon source, high-purity graphite substrate was used as the carbon source, and SiCnws were obtained by heating to 1350 °C under Ar protective atmosphere. SiCnws of 250D and 450D were provided by Changsha Snett Advanced Materials Co., Ltd. in Changsha, China. The 6061 aluminum alloy was provided by Northeast Light Alloy Co., Ltd., in Harbin, China. The chemical composition of 6061Al alloy is 0.24 wt.% Cu, 1.01 wt.% Mg, 0.15 wt.% Mn, 0.70 wt.% Fe, 0.64 wt.% Si, 0.25 wt.% Zn, 0.29 wt.% Cr and else Al.

**Figure 1:**
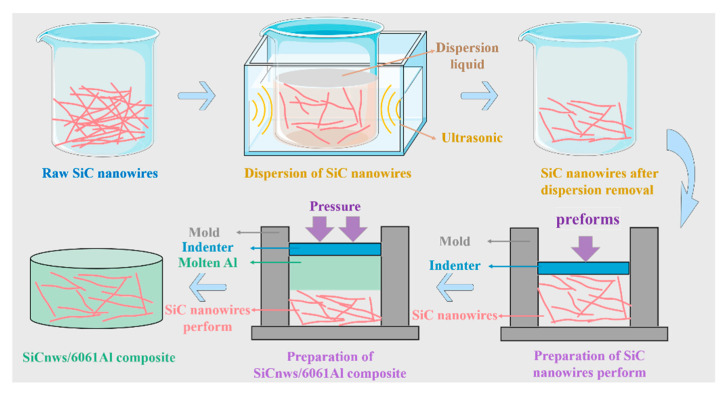
The schematic diagram of preparation of SiCnws/6061Al.

The manufacturing process of SiCnws/6061Al composites is similar to that of previous composites. Composites are prepared by pressure infiltration technology [24]. As shown in Figure 1, the composites obtained by using 100D, 250D and 450D SiCnws are named 100D SiCnws/6061Al, 250D SiCnws/6061Al and 450D SiCnws/6061Al, respectively. The SiCnw was put into a steel mold and further pressed to prepare the preforms, then was heated to 550 °C at a rate of 5 °C/min. The volume content of the milled particles in the mold was about 60 vol%. The preheating temperatures of preform and graphite indenter are 550 °C and 780 °C, respectively. The 10 MPa pressure was maintained for 7 min during the infiltration process. Prior to mechanical testing, the specimens were subjected to peak-aging treatment (530 °C/1.5 h solution, quenched in water, 175 °C/6 h aging).

### 2.2. Microstructure Characterization and Mechanical Properties Testing

The microstructure and morphology of the raw material SiCnws were characterized by a field emission scanning electron microscope (SEM, HELIOS NanoLab 600i). Field emission transmission electron microscopy (TEM, JEM-2010F, JEOL, Tokyo, Japan) was used to observe the microstructure of the composites. X-ray diffractometer (XRD, Rigaku D/max-rB) to detect phase composition, using Cu-Ka radiation for identification, scanning angle of 25° to 90°, scanning speed of 3°/min, tube voltage 40 Kv, tube current 40 mA. The polished samples were etched with 10 vol.% NaoH for 60 s and then observed by SUPRA55 scanning electron microscope (SEM, Zeiss Corporation, Oberkochen, Germany).

At the same time, local composition analysis was performed by energy dispersive spectroscopy (EDX). The sample density was measured using the Archimedes principle. The mass of the samples placed in air and water was measured by a precision balance. The formula for calculating the density is as follows:

$$\rho =\frac{m_{Air}}{\left(m_{Air}-m_{H_{2}O}\right)/\rho_{H_{2}O}}$$

**Figure 2:**
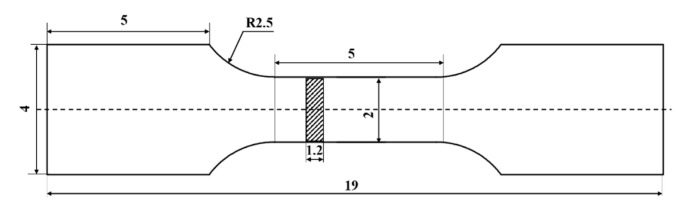
Structure diagram of tensile samples.

In order to improve the significance of the statistical results, five samples were tested each. By comparing the measured true density and the theoretical density of the sample, the relative density of the composite material is calculated. Tensile samples were tested on Instron 5569 universal electrical tensile testing machine, and the size of the tensile samples was shown in Figure 2. Finally, SEM was used to observe the fracture of the composites.

## 3. Results and Discussion

**Figure 3:**
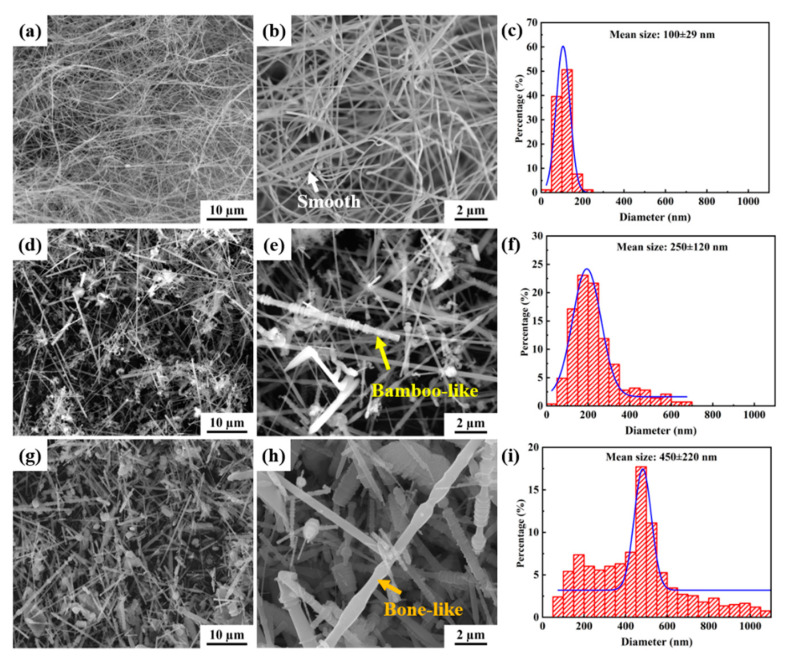
SEM and diameter distribution images of SiCnws. (**a**,**b**,**d**,**e**,**g**,**h**) Morphology of 100D, 250D and 450D SiCnws, respectively; (**c**,**f**,**i**) Diameter distribution of 100D, 250D and 450D SiCnws, respectively.

The morphology of the SiCnw is characterized by SEM, as shown in Figure 3. The length and diameter of the 100D SiC nanowires range from 50 to 100 μm (Figure 3a) and 40 to 250 nm (Figure 3b), respectively, with an average diameter of 100 nm (Figure 3c), exhibiting a curved morphology. The length and diameter distribution of 250D SiC nanowires are 10~80 μm (Figure 3d) and 50~700 μm (Figure 3e), respectively, and the average diameter is 250 nm (Figure 3f). The length and diameter distributions of the 450D SiC nanowires used are 5–50 μm (Figure 3d) and 50–1100 μm (Figure 3e), respectively, with an average diameter of 450 nm (Figure 3f).

**Figure 4:**
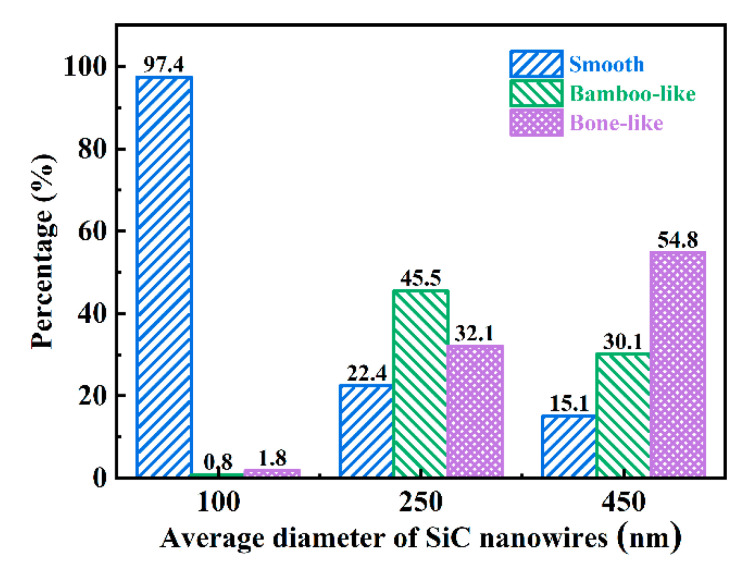
Statistical results of SiCnws’ morphology.

Three kinds of SiC morphologies were observed. They are cylindrical with uniform diameter and a smooth surface (as shown by the white arrows in Figure 3b), bamboo-like with a rough surface of diameter fluctuation (yellow arrows in Figure 3e) and bone-like with a smooth surface of diameter fluctuation (orange arrows in Figure 3h). The surface roughness is related to the size of the nanowires; the larger the surface, the rougher it is. The surface of 100D is smoother, while the surfaces of 250D and 450D nanowires have more protrusions, which are bamboo-like and bone-like. Although nanowires have different morphologies, studies have shown that they all have the effect of inducing stacking faults [38]. It is worth noting that the proportion of each morphology is different. Figure 4 shows the statistics of the three morphologies in SiC nanowires with different diameters. In the nanowires with an average diameter of 100 nm, the proportion of cylindrical, bamboo and bone rod are 97.4%, 0.8% and 1.8%; the ratio of three morphologies of 250 nm is 22.4%, 45.5% and 32.1%; the morphology of 450 nm nanowires accounted for 15.1%, 30.1% and 54.8%, respectively. It is worth noting that there is a certain correlation between the morphology and diameter of SiCnws. When the diameter is small, it exhibits a smooth cylindrical surface, and when the nanowire diameter is large, it is easier to exhibit bamboo-like and bone-like surfaces [39,40,41].

**Figure 5:**
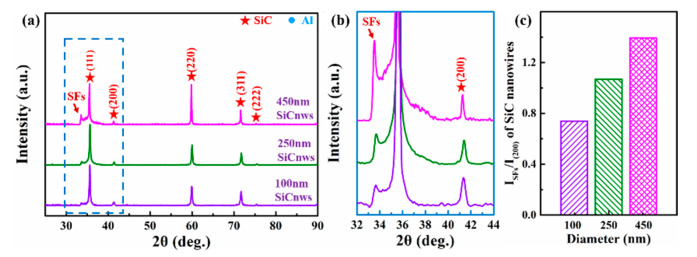
The XRD patterns of the raw SiCnws. (**a**) Overall patterns and the high magnification of the (**b**) 32°–44° of the SiCnws, (**c**) The SFs/(200) peak value ratio of the SiCnws.

Figure 5 is the XRD pattern of SiC nanowires. There are diffraction peaks at 33.7°, 35.7°, 41.4°, 60.0°, 71.8° and 75.6°. Using the standard powder XRD pattern (JCPDS card), 35.7°, 41.4°, 60.0°, 71.8° and 75.6° correspond to 3C-SiC (JCPDS No.29-1129; (111), (200), (220), (311) and (222) crystal planes of a = 4.359 Å, and space group F-43 m) [42]. According to previous research [43], the diffraction peak at 33.7° is caused by stacking faults on the (111) surface of 3C-SiC. Chen et al. [44] evaluated the stacking fault density (*X*) by the intensity ratio of the SFs peak to the SiC (200) peak, as shown in Equation (1):

(1) $$X=\frac{I_{\mathrm{SFs}}}{I_{\mathrm{SiC}\;(200)}}$$

*I*_SFs_ and *I*_SiC (200)_ are the intensity values of SFs and SiC (200) peaks, respectively. The larger *X* indicates the higher stacking-fault density in SiC. Figure 5b is a local enlarged figure of 32°–44°, and the stacking fault densities of 100 nm, 250 nm and 450 nm (Figure 5c) are 0.74, 1.07 and 1.40, respectively. Therefore, the defect content of SiCnws with different diameters can be divided into 100D < 250D < 450D.

**Figure 6:**
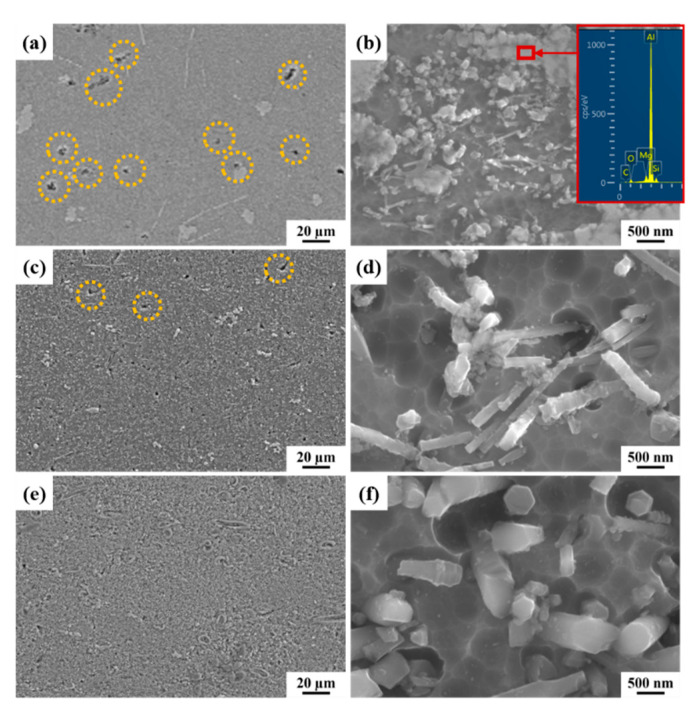
The representative SEM microstructure of SiCnw/6061Al composites. (**a**,**b**) 100D SiCnws/6061Al, (**c**,**d**) 250D SiCnws/6061Al, (**e**,**f**) 450D SiCnws/6061Al.

The microstructure of SiCnws/6061Al composites is shown in Figure 6. A large number of small holes were found in 100D SiCnws/6061Al (orange dotted box in Figure 6a), which was due to poor wettability between SiC and Al [45]. Yin et al. [45] found that the amount of added β-Si_3_N_4_ whiskers and sintering temperature had a significant effect on the density of the composites. Zhang et al. [46] found that the surface modification of β-Si_3_N_4_ whiskers can improve the relative density of β-Si_3_N_4_w/Al composites. Feng et al. [47] electroless plated Cu on the surface of SiCp, which could greatly improve the wettability of SiCp and Al-Si alloys, and then the density of the composites was greatly improved. These results can be used to further improve the densification results of the material.

In this study, as the diameter of the nanowires increases to 250 nm, the number of holes in the composite decreases (Figure 6c, orange virtual coil). When the diameter of SiCnws further increases to 450 nm, no holes are found (Figure 6e), indicating that the increase in the diameter of SiCnws increases the density of the composite. It can be observed in Figure 6b that nanowires are uniformly distributed in the composite, and the protrusions are uncorroded Al matrix (red box in Figure 6b) by EDX analysis. It is worth noting that, after NaOH etching, more aluminum adheres to the nanowires with rough surfaces (Figure 6d), while almost no residual aluminum is observed on the smooth nanowires (Figure 6f), which can be attributed to the difference in interface bonding and interface area [22].

**Figure 7:**
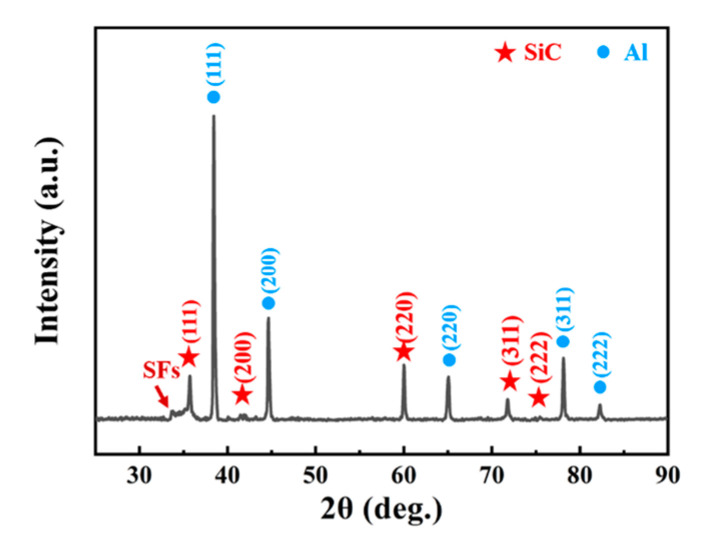
The XRD pattern of the SiCnws/6061Al composite.

The XRD patterns of typical SiCnws/6061Al composites are shown in Figure 7. In addition to the characteristic peak of SiC, there are diffraction peaks at 38.5°, 44.74°, 65.13°, 78.23° and 82.44°, corresponding to Al (JCPDS No.04-0787 (111), (200), (220), (311) and (222) planes of a = 4.0494 Å, and space group F-3 m).

**Figure 8:**
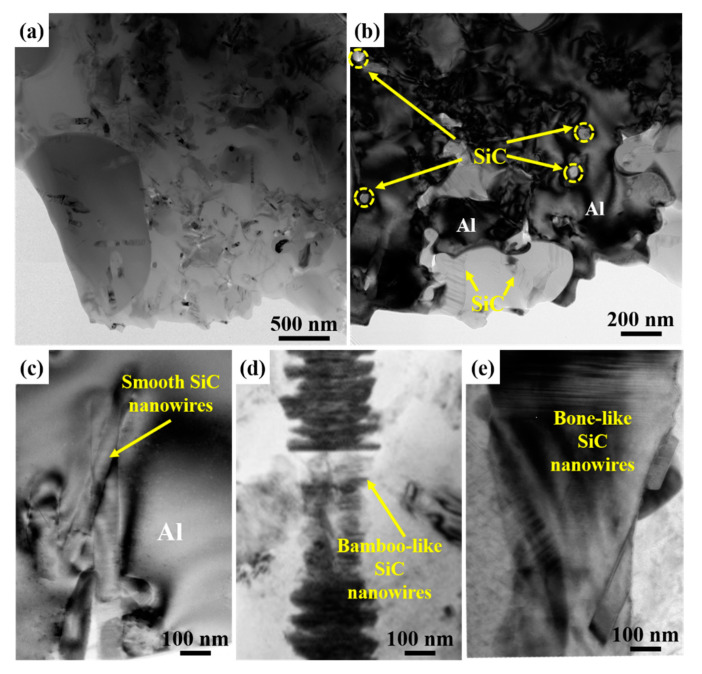
The TEM microstructure of SiCnws/6061Al composite. (**a**) Low magnification image of 100D SiCnws/6061Al, (**b**) SiC nanowires located within the Al grains in the 100D SiCnws/6061Al, (**c**) Smooth SiC nanowires in the 100D SiCnws/6061Al, (**d**) Bamboo-like SiC nanowires in the 250D SiCnws/6061Al, (**e**) Bone-like SiC nanowires in the 450D SiCnws/6061Al.

The microstructure of SiCnws/6061Al composites was further observed by TEM, as shown in Figure 8. SiCnws with a diameter of 100 nm are uniformly and randomly distributed in Al (Figure 8a), and most of the SiCnws are located inside the Al grains (Figure 8b, yellow virtual coil). In the process of SiCnws/Al composites prepared by pressure infiltration, SiCnws are mainly distributed in the Al grains due to the rapid solidification of the Al matrix [24]. At the same time, the composite has a smaller grain size of about 200 nm to 1 μm, and there are a large number of tangled dislocations in the aluminum grains (Figure 8b). The nanophase (such as nanotubes, nanowires and nanoplates) inhibits the growth of the matrix grains and greatly improves the fine-grain strengthening effect of the composites [13,15,48,49]. Due to the large difference in thermal expansion coefficient between SiCnws and Al, a large number of dislocations are generated, and the pinning effect of SiCnws on the interface refines the grains of the Al matrix. At the same time, in the composites, smooth (Figure 8c), bamboo-like (Figure 8d) and bone-like (Figure 8e) SiCnws have good bonding with Al, the interface is clean and no interfacial reaction products are found.

**Figure 9:**
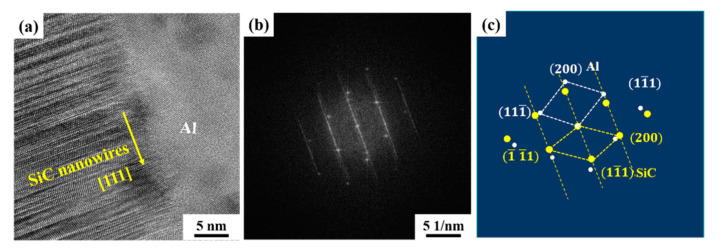
The HRTEM image of SiCnws/6061Al composite. (**a**) The interface between the SiCnws and Al matrix, (**b**) Corresponding SEAD patterns, (**c**) Calibration results of SEAD pattern in (**b**).

The interfacial microstructure between SiC nanowires and the Al matrix was further observed by HRTEM, as shown in Figure 9. Selective electron diffraction patterns and their calibration results at the SiC/Al interface were given in Figure 9b,c, respectively. The calibration results showed that there were only diffraction spots of SiC and Al, which were similar to the results of micron SiC particles [50], nano SiC particles [51] and graphene [48] reinforced aluminum matrix composites prepared by pressure infiltration, and no interface products (such as Al_4_C_3_, SiO_2_ or Al_2_O_3_) were found [52,53,54,55], which showed that SiC nanowires were not damaged during the preparation process.

**Figure 10:**
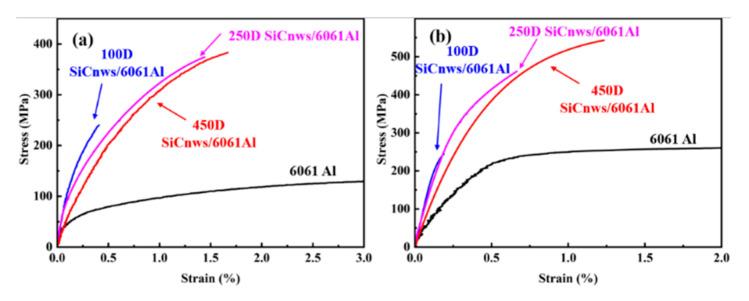
The tensile curves of the representative 6061Al, 20 vol.% 100D, 250D and 450D SiCnw/6061Al composites. (**a**) Annealed, (**b**) Peak-aged.

**Figure 11:**
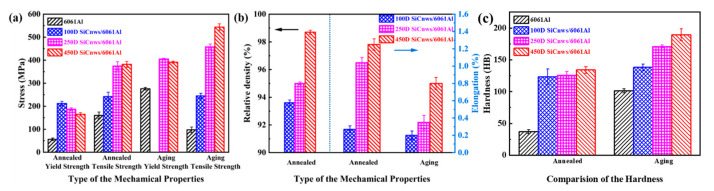
The tensile performance and relative density of 6061Al, 20vol.% 100D, 250D and 450D SiCnw/6061Al composites. (**a**) Comparison of the yield and tensile strength, (**b**) Comparison of the relative density and elongation, (**c**) Hardness.

The tensile curves of representative annealed and aged 20 vol.% 100D, 250D and 450D SiCnws/6061Al composites are shown in Figure 10. Compared with the 6061Al matrix, the strength of the composites has been greatly improved. With the increase in the diameter of the reinforced phase, the strength and plasticity of SiCnws/6061 Al composites increase. SiCnws/6061Al composites after annealing show better plastic deformation than peak aging. The specific tensile properties and relative density of the composites are shown in Figure 11. Without considering the heat treatment, the fracture strength (Figure 11a), relative density and elongation (Figure 11b) increase with the increase in nanowire diameter. The hardness test results are shown in Figure 11c. It can be seen that the hardness of the composites increases with the increase in SiCnw diameter. With the decrease in the nanowire size, the infiltration of the composite material becomes more difficult, which inevitably leads to an increase in defects in the composite material. Since the peak-aged 100D SiCnws/6061Al composites fractured before yielding, the yield strength was not obtained. However, in the annealed state, the yield strength decreased with the increase in nanowire diameter.

**Figure 12:**
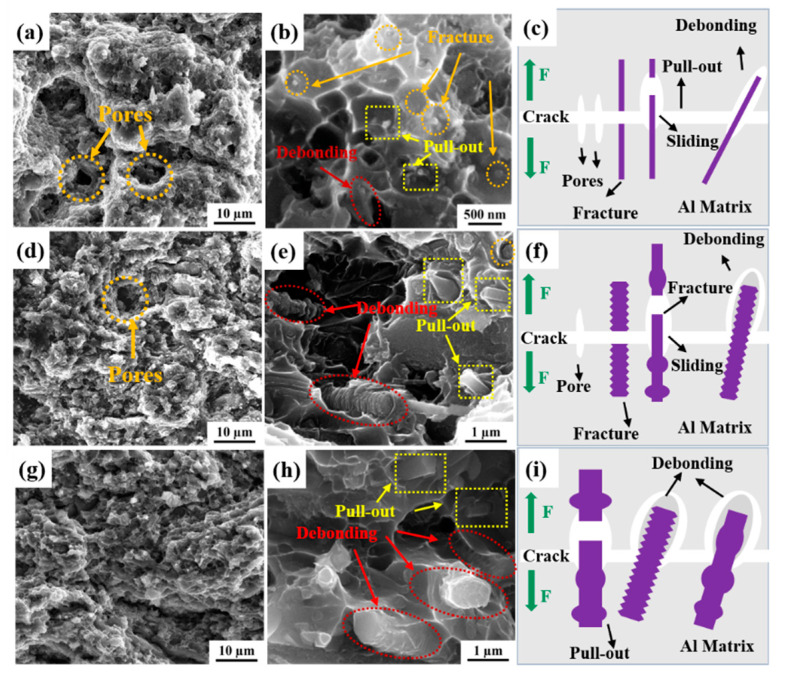
Fracture morphology of SiCnw/6061Al composites prepared by SiCnws with different diameters. (**a**,**b**) 100 nm, (**c**) Schematic corresponding to (**a**–**d**) 250 nm, (**f**) Schematic corresponding to (**d**,**e**), (**g**,**h**) 450 nm, (**i**) Schematic corresponding to (**g**,**h**).

The fracture morphology of 20 vol.% 100D, 250D and 450D SiCnws/6061Al composites is shown in Figure 12. Holes can be found in the fracture of 100D SiCnws/6061Al composites (Figure 12a, orange box). The fracture of nanowires is mainly divided into three characteristics: negligible pull-out fracture (Figure 12b, orange circle), obvious pull-out fracture (Figure 12b, yellow box) and nanowire/Al debonding (Figure 12b, red oval), in which the pull-out fracture is mainly neglected. At the same time, a small amount of the Al matrix is observed on the smooth surface of SiCnws, and the fracture diagram is shown in Figure 12c. There are a few pores in the fracture of 250D SiCnw/6061Al composites (Figure 12d), and the fracture characteristics of nanowires in 100D SiCnws/6061Al are divided into three types (Figure 12e). However, due to the high content of bamboo-like and rod-like nanowires (45.5% and 32.1%, respectively), the fracture morphology of SiCnws is mainly characterized by the obvious pull-out fracture (Figure 12e, yellow box) and nanowire/Al debonding (Figure 12e, red ellipse). It is worth noting that there is still a large amount of adhesive Al on the surface of bamboo-like SiCnws, and the fracture diagram is shown in Figure 12f. No obvious holes were found in the fracture of 450D SiCnws/6061Al. 30.1% bamboo-like and 54.8% bone rod-like in the raw materials, making the fracture morphology of SiCnws mainly obvious pull-out fracture (Figure 12h, yellow box) and nanowire/Al debonding (Figure 12h, red ellipse). Similarly, a large amount of Al matrix is also bonded on the surface of bone rod-like SiCnws, and the fracture diagram is shown in Figure 12i.

The properties of composites are closely related to density and interfacial bonding strength [37]. It can be seen that the 100D SiCnw/6061Al composite has the lowest density (93.6%). Therefore, in the mechanical properties test, more holes make the crack initiate rapidly. At the same time, because the interface between SiCnws and Al is mechanically bonded (Figure 9a), the bonding strength between SiCnws with a smooth surface and the aluminum matrix is weak, resulting in rapid instability and early fracture of the material (Figure 12c), showing low strength and low plasticity in mechanical properties. The density of 450D SiCnw/6061Al composites is the highest, reaching 98.9%. At the same time, due to the higher interlocking effect of the bamboo-like morphology than nanowires with a smooth surface, the average interface strength of 450D SiCnw/6061Al composites is higher than that of 100D SiCnw/6061Al composites, and the crack is difficult to deflect at the interface. Therefore, the former has a similar plastic shape to the latter, but has higher strength. The improvement in plasticity and strength of 250D SiCnw/6061Al composites compared with 100D SiCnw/6061Al composites can be attributed to the improvement in interface bonding and the increase in density.

Previous studies have shown that SiCnWs with non-uniform diameters are more uniform than nanowires with uniform diameters, which can produce a strong interlocking effect with the matrix. SiCnws are not easy to pull out, which can make more effective use of the bearing effect of the reinforcement and help to improve the strength and deformation of the matrix [22,56]. For ceramic-reinforced composites, when the reinforcement size is large, cracks at the reinforcement or interface are easily initiated and propagated, resulting in a significant reduction in the ductility and toughness of the material [33,57]. As a result, 250D SiCnws/6061Al composite has better plasticity than 450D SiCnw/6061Al composite, which can be attributed to the higher content of bamboo-like nanowires in 250D SiCnw/6061Al composite. The interlocking effect at the interface is enhanced, and the smaller size makes the crack at the interface not easy to initiate and propagate.

The yield strength is usually less dependent on stress concentration and is more representative for discussing strengthening behavior. The improved shear-lag model can explain the strengthening behavior of SiCnws/Al composites well [20,24]. When the length of nanowires (*l*) is greater than the critical length (*l*_c_), where lc is usually 5–10 times the diameter of nanowires (*d*), the yield strength (*σ*_cy_) of SiCnws/Al composites can be expressed as Equation (2):

(2) $$\sigma_{\mathrm{cy}}{=\sigma }_{\mathrm{my}}+\mathrm{ckg}\psi \frac{l}{d}\left(2-\frac{l_{c}}{l}\right)\tau_{\max}V_{r}$$

*σ*_my_ is the yield strength of the matrix, *c* is the modified empirical constant, *k* is the matrix-reinforcement interface bonding performance, the geometric factor *g* is the surface volume ratio, the alignment factor *ψ* is the average angle between the loading direction and the nanowires, *τ*_max_ is the maximum shear stress, and *V*_r_ is the volume fraction of the reinforcing phase. In the as-cast composite, SiCnws are randomly distributed (*ψ* is the same). The increase in the specific surface area *g* of the reinforcement effectively improves the yield strength *σ*_cy_.

According to Equation (2), in order to further utilize the strengthening effect of SiCnws, in addition to reducing the size of nanowires, further research can be carried out in the following fields: (1) Optimizing the interface structure to further enhance the interface bonding strength, such as adding alloying elements [58], interface modification [46,59] and increasing the bamboo-like content [15,22]. (2) When the reinforcements are randomly distributed and aligned, the orientation optimization of SiCnws shows that the alignment factor ψ is 0.56~0.66 and 1, respectively, [37]. By extrusion, quasi-aligned nanowires can be obtained. However, traditional extrusion leads to severe fracture of nanowires, which offsets the strengthening effect. For this study, the low density of the composite material seriously limits the strengthening effect of SiCnws. Therefore, further improvements to the density of the material and the quasi-directional arrangement of the nanowires while ensuring the integrity of the nanowires will become the focus of the next research.

## 4. Conclusions

In the present work, the effects of SiCnws with diameters of 100 nm, 250 nm and 450 nm on the microstructure and mechanical behavior of 20 vol.% SiCnws/6061Al composites prepared by pressure infiltration were studied. The following conclusions were obtained:

1. SEM and TEM analysis showed that SiCnws were uniformly distributed in the SiCnws/6061Al composite. TEM observation showed that the interface between SiCnws and the Al matrix was well bonded and no interface product was found in the composite.

2. With the diameter of the reinforcement increased, the density of the composite increased, and the composite with 450 nm SiCnws reached 98.9%. The tensile strength and plasticity of SiCnws/6061Al composites increased with an improvement in the average diameter of SiCnws. The tensile strength of the composites prepared by 450 nm SiCnws reached 544 MPa and the elongation reached 1.25%, which could be attributed to the increase in density and the improvement in interface bonding.

3. With the decrease in the diameter of the nanowires, the effective atoms involved in the interface load transfer increased, and the strengthening effect of SiCnws increased. Under the annealing state, the yield strength of the SiCnws/6061Al composite prepared by 100 nm reached 212 MPa, which was 13.4% and 28.5% higher than that of 250 nm and 450 nm, respectively.

4. The fracture analysis showed that the fracture characteristics of nanowires in smooth SiCnws-reinforced composites were dominated by negligible pull-out fractures. In bamboo-like and rod-like SiCnws-reinforced composites, the characteristics of nanowires were dominated by obvious pull-out fractures and nanowire/Al debonding.

## Data Availability

The data presented in this study are available on request from the corresponding author.

## References

[1] Jin Y., Zhang B., Zhang H., Zhong Z., Wang Y., Ye F., Liu Q. (2021). Effects of skeleton pore size on the microstructure and electromagnetic absorbing property of the SiC nanowires/SiC composites. Mater. Lett..

[2] Zhang Q., Zeng C., Wu Z., Xie Z., Zou Y., Chen D. (2019). Preparation of beaded chains ZrC/C/SiC nanocomposites and their microwave absorption properties. Mater. Lett..

[3] Zhou C., Zhou Y.X., Liu S.Y., Zhang L.Y., Song P.Y., Zhang Q., Ma B.Y., Wu G.H. (2022). Lightweight and near-zero thermal expansion ZrW_2_O_8_-SiCnw/Al hybrid composites. J. Alloys Compd..

[4] Li X., Lu X.K., Li M.H., Xue J.M., Ye F., Fan X.M., Liu Y.S., Cheng L.F., Zhang L.T. (2022). A SiC nanowires/Ba_0.75_Sr_0.25_Al_2_Si_2_O_8_ ceramic heterojunction for stable electromagnetic absorption under variable-temperature. J. Mater. Sci. Technol..

[5] Fan J., Xu S. (2018). Thermal conductivity and mechanical properties of high density polyethylene composites filled with silicon carbide whiskers modified by cross-linked poly (vinyl alcohol). J. Mater. Sci. Technol..

[6] Fu Q.-G., Peng H., Nan X.-Y., Li H.-J., Chu Y.-H. (2014). Effect of SiC nanowires on the thermal shock resistance of joint between carbon/carbon composites and Li_2_O–Al_2_O_3_–SiO_2_ glass ceramics. J. Eur. Ceram. Soc..

[7] Zhu Q., Dong X., Hu J.B., Yang J.S., Zhang X.Y., Ding Y.S., Dong S.M. (2020). High strength aligned SiC nanowire reinforced SiC porous ceramics fabricated by 3D printing and chemical vapor infiltration. Ceram. Int..

[8] Ruan J., Yang J., Dong S., Yan J., Zhang X., Ding Y., Zhou H., Hu J. (2019). Interfacial optimization of SiC nanocomposites reinforced by SiC nanowires with high volume fraction. J. Am. Ceram. Soc..

[9] Chong X., Xiao G., Ding D., Luo J., Zheng X. (2022). Combustion synthesis of SiC/Al_2_O_3_ composite powders with SiC nanowires and their growth mechanism. Ceram. Int..

[10] Tang M., Liu Y., Liu L., Lin T., Liu X. (2021). Microstructure and mechanical properties of SiC/SiC joints reinforced by in-situ growth SiC nanowires. Mater. Charact..

[11] Xie C., Song S., He G., Mao Z., Ma C., Zhang T., Hu P., Zhen Q. (2022). The toughening design of multi-layer antioxidation coating on C/C matrix via SiC-SiCw transition layer grown in-situ. J. Eur. Ceram. Soc..

[12] Qian J., Shui A., He C., Wang X., Cai M., Pu Y., Hu P., Du B. (2021). Multifunction properties of SiOC reinforced with carbon fiber and in-situ SiC nanowires. Ceram. Int..

[13] Li W., Nie K.B., Liu Z.L., Deng K.K., Li Y.A., Tong X.L. (2022). Effect of hot extrusion on the microstructure and mechanical properties of SiCNWs/Mg-2Zn-0.1Y composite. Mater. Charact..

[14] Zhang T., Qi L., Fu J., Zhou J., Chao X. (2019). Effect of SiC nanowires addition on the interfacial microstructure and mechanical properties of the Cf-SiCNWs/AZ91D composite. J. Alloys Compd..

[15] Liu Y., Dong L.L., Lu J.W., Huo W.T., Du Y., Zhang W., Zhang Y.S. (2020). Microstructure and mechanical properties of SiC nanowires reinforced titanium matrix composites. J. Alloys Compd..

[16] Li S., Zhang Y., Han J., Zhou Y. (2013). Fabrication and characterization of SiC whisker reinforced reaction bonded SiC composite. Ceram. Int..

[17] Komai K., Minoshima K., Ryoson H. (1993). Tensile and fatigue fracture behavior and water-environment effects in a SiC-whisker/7075-aluminum composite. Compos. Sci. Technol..

[18] Jintakosol T., Kumfu S., Singjai P., Busabok C. (2012). Effect of Wear Tests on Silicon Carbide Nanowires/Aluminium Metal Powder Composites. Chiang Mai J. Sci..

[19] Yang W., Dong R., Yu Z., Wu P., Hussain M., Wu G. (2015). Strengthening behavior in high content SiC nanowires reinforced Al composite. Mater. Sci. Eng., A.

[20] Xin L., Yang W., Zhao Q., Dong R., Wu P., Xiu Z., Hussain M., Wu G. (2017). Strengthening behavior in SiC nanowires reinforced pure Al composite. J. Alloys Compd..

[21] Dong R., Yang W., Yu Z., Wu P., Hussain M., Jiang L., Wu G. (2015). Aging behavior of 6061Al matrix composite reinforced with high content SiC nanowires. J. Alloys Compd..

[22] Dong R., Yang W., Wu P., Hussain M., Yu Z., Jiang L., Wu G. (2015). Effect of reinforcement shape on the stress-strain behavior of aluminum reinforced with SiC nanowire. Mater. Des..

[23] Dong R., Yang W., Wu P., Hussain M., Wu G., Jiang L. (2015). High content SiC nanowires reinforced Al composite with high strength and plasticity. Mater. Sci. Eng. A.

[24] Dong S., Zhang B., Zhan Y., Liu X., Xin L., Yang W., Wu G. (2019). Effect of Extrusion Temperature on the Microstructure and Mechanical Properties of SiCnw/2024Al Composite. Materials.

[25] Zhang Q., Ma X., Wu G. (2013). Interfacial microstructure of SiCp/Al composite produced by the pressureless infiltration technique. Ceram. Int..

[26] Adin M.S., Kilickap E. (2021). Strength of double-reinforced adhesive joints. Mater Test.

[27] Adin H., Adin M.S. (2022). Effect of particles on tensile and bending properties of jute epoxy composites. Mater Test.

[28] Kumar V.M., Venkatesh C.V. (2019). A comprehensive review on material selection, processing, characterization and applications of aluminium metal matrix composites. Mater. Res. Express.

[29] Mousavian R.T., Khosroshahi R.A., Yazdani S., Brabazon D., Boostani A.F. (2016). Fabrication of aluminum matrix composites reinforced with nano- to micrometer-sized SiC particles. Mater. Des..

[30] Zhang S., Chen Z., Wei P., Liu W., Zou Y., Lei Y., Yao S., Zhang S., Lu B., Zhang L. (2022). Wear properties of graphene/zirconia biphase nano-reinforced aluminium matrix composites prepared by SLM. Mater. Today Commun..

[31] Surakasi R., Rajendra Prasad A., Pattnaik B., Venkata Rao M., Puthilibai G., Vibhakar C., Rajkumar S. (2022). Mechanical behaviour of nano ceramic particles reinforced aluminium matrix composites. Mater. Today Proc..

[32] Pragathi P., Elansezhian R. (2022). Studies on microstructural and mechanical properties of (Nano SiC + Waste Spent catalyst) reinforced aluminum matrix composites. Mater. Today Commun..

[33] Padmavathi K.R., Ramakrishnan R., Karthikeyan L., Tamizhselvan S., Chezhian Babu S. Comparison of the mechanical properties of micro/nano SiC/TiO_2_ reinforced aluminium metal matrix composites. Mater. Today. Proc..

[34] Shirasu K., Nakamura A., Yamamoto G., Ogasawara T., Shimamura Y., Inoue Y., Hashida T. (2017). Potential use of CNTs for production of zero thermal expansion coefficient composite materials: An experimental evaluation of axial thermal expansion coefficient of CNTs using a combination of thermal expansion and uniaxial tensile tests. Compos. Part A-Appl. Sci. Manuf..

[35] Zhou W., Bang S., Kurita H., Miyazaki T., Fan Y., Kawasaki A. (2016). Interface and interfacial reactions in multi-walled carbon nanotube-reinforced aluminum matrix composites. Carbon.

[36] Singla D., Amulya K., Murtaza Q. (2015). CNT reinforced Aluminium matrix Composite-a review. Mater. Today-Proc..

[37] Zhang C.X., Zeng Y.P., Yao D.X., Yin J.W., Zuo K.H., Xia Y.F., Liang H.Q. (2019). The improved mechanical properties of Al matrix composites reinforced with oriented beta-Si_3_N_4_whisker. J. Mater. Sci. Technol..

[38] Dong R., Yang W., Wu P., Hussain M., Xiu Z., Wu G., Wang P. (2015). Microstructure characterization of SiC nanowires as reinforcements in composites. Mater. Charact..

[39] Shen Z., Chen J., Li B., Li G., Zheng H., Men J., Hou X. (2020). Tunable fabrication and photoluminescence property of SiC nanowires with different microstructures. Appl. Surf. Sci..

[40] Chen S., Li W., Li X., Yang W. (2019). One-dimensional SiC nanostructures: Designed growth, properties, and applications. Prog. Mater Sci..

[41] Liu C., Yuan X., Wang W., Liu H., Li C., Wu H., Hou X. (2022). In-situ fabrication of ZrB_2_-ZrC-SiCnws hybrid nanopowders with tuneable morphology SiCnws. Ceram. Int..

[42] Wang X., Yang T., Hou X., Zheng Y., Wang E., Du Z., Cao S., Wang H. (2022). Preparation of 2H/3C–SiC heterojunction nanowires from molten salt method with blue shift photoluminescence property. Ceram. Int..

[43] Lodhe M., Balasubramanian M. (2022). Polycarbosilane facilitated growth of SiC nanowires from biowaste coconut shell. Adv. Appl. Ceram..

[44] Chen B.-Y., Chi C.-C., Hsu W.-K., Ouyang H. (2021). Synthesis of SiC/SiO_2_ core-shell nanowires with good optical properties on Ni/SiO_2_/Si substrate via ferrocene pyrolysis at low temperature. Sci. Rep..

[45] Yin J., Yao D., Hu H., Xia Y., Zuo K., Zeng Y.-P. (2014). Improved mechanical properties of Cu matrix composites reinforced with β-Si_3_N_4_ whiskers. Mater. Sci. Eng. A.

[46] Zhang C., Yao D., Yin J., Zuo K., Xia Y., Liang H., Zeng Y.-P. (2018). Microstructure and mechanical properties of aluminum matrix composites reinforced with pre-oxidized β-Si_3_N_4_ whiskers. Mater. Sci. Eng. A.

[47] Feng Y., Ren J.P., Dong C.G., Peng C.Q., Wang R.C. (2017). Microstructures and Properties of the Copper-Coated SiCp Reinforced Al-Si Alloy Composites. Adv. Eng. Mater..

[48] Yu Z., Yang W., Zhou C., Zhang N., Chao Z., Liu H., Cao Y., Sun Y., Shao P., Wu G. (2019). Effect of ball milling time on graphene nanosheets reinforced Al6063 composite fabricated by pressure infiltration method. Carbon.

[49] Wang J., Li Z., Fan G., Pan H., Chen Z., Zhang D.J.S.M. (2012). Reinforcement with graphene nanosheets in aluminum matrix composites. Scr. Mater..

[50] Cui Y., Jin T., Cao L., Liu F. (2016). Aging behavior of high volume fraction SiCp/Al composites fabricated by pressureless infiltration. J. Alloys Compd..

[51] Yang W., Dong R., Jiang L., Wu G., Hussain M. (2015). Unstable stacking faults in submicron/micron Al grammins in multi-SiCp/multi-Al nanocomposite. Vacuum.

[52] Guo B., Zhang X., Cen X., Wang X., Song M., Ni S., Yi J., Shen T., Du Y. (2018). Ameliorated mechanical and thermal properties of SiC reinforced Al matrix composites through hybridizing carbon nanotubes. Mater. Charact..

[53] Zhu J., Wang F., Wang Y., Zhang B., Wang L. (2017). Interfacial structure and stability of a co-continuous SiC/Al composite prepared by vacuum-pressure infiltration. Ceram. Int..

[54] Wang Z.G., Li C.P., Wang H.Y., Zhu X., Wu M., Jiang Q.C. (2016). Effect of nano-SiC content on mechanical properties of SiC/2014Al composites fabricated by powder metallurgy combined with hot extrusion. Powder Metall..

[55] Zhou W., Yamaguchi T., Kikuchi K., Nomura N., Kawasaki A. (2017). Effectively enhanced load transfer by interfacial reactions in multi-walled carbon nanotube reinforced Al matrix composites. Acta Mater..

[56] Pozuelo M., Kao W.H., Yang J.M. (2013). High-resolution TEM characterization of SIC nanowires as reinforcements in a nanocrystalline Mg-matrix. Mater. Charact..

[57] Zhou D.S., Qiu F., Wang H.Y., Jiang Q.C. (2014). Manufacture of Nano-Sized Particle-Reinforced Metal Matrix Composites: A Review. Acta Metall. Sin.-Engl. Lett..

[58] Yang W., Chen G., Qiao J., Zhang Q., Dong R., Wu G. (2017). Effect of Mg addition on the microstructure and mechanical properties of SiC nanowires reinforced 6061Al matrix composite. Mater. Sci. Eng., A.

[59] Gao Y.-Y., Qiu F., Geng R., Zhao W.-X., Yang D.-L., Zuo R., Dong B.-X., Han X., Jiang Q.-C. (2018). Preparation and characterization of the Al-Cu-Mg-Si-Mn composites reinforced by different surface modified SiCp. Mater. Charact..

